# Melatonin Enhances Neural Differentiation of Adipose-Derived Mesenchymal Stem Cells

**DOI:** 10.3390/ijms25094891

**Published:** 2024-04-30

**Authors:** Ivana Roberta Romano, Floriana D’Angeli, Elisa Gili, Mary Fruciano, Giuseppe Angelo Giovanni Lombardo, Giuliana Mannino, Nunzio Vicario, Cristina Russo, Rosalba Parenti, Carlo Vancheri, Rosario Giuffrida, Rosalia Pellitteri, Debora Lo Furno

**Affiliations:** 1 Department of Biomedical and Biotechnological Sciences, University of Catania, 95123 Catania, Italy; ivanarobertaromano@yahoo.it (I.R.R.); nunzio.vicario@unict.it (N.V.); cristina.russo@unict.it (C.R.); parenti@unict.it (R.P.); giuffros@unict.it (R.G.); lofurno@unict.it (D.L.F.); 2 Department of Human Sciences and Quality of Life Promotion, San Raffaele Roma Open University, 00166 Rome, Italy; floriana.dangeli@uniroma5.it; 3 Department of Clinical and Experimental Medicine, University of Catania, 95124 Catania, Italy; elisa.gili@unict.it (E.G.); mary.fruciano@unict.it (M.F.); vancheri@unict.it (C.V.); 4 Faculty of Medicine and Surgery, University of Enna “Kore”, 94100 Enna, Italy; giuseppe.lombardo@unikore.it; 5 Cannizzaro Hospital, 95126 Catania, Italy; 6 Department of Chemical, Biological, Pharmaceutical and Environmental Sciences, University of Messina, 98122 Messina, Italy; 7 Institute for Biomedical Research and Innovation, National Research Council, 95126 Catania, Italy; rosaliamariacristina.pellitteri@cnr.it

**Keywords:** adipose-derived mesenchymal stem cells, melatonin, glial conditioned media, neuronal differentiation, olfactory ensheathing cells, Schwann cells, melatonin receptors

## Abstract

Adipose-derived mesenchymal stem cells (ASCs) are adult multipotent stem cells, able to differentiate toward neural elements other than cells of mesodermal lineage. The aim of this research was to test ASC neural differentiation using melatonin combined with conditioned media (CM) from glial cells. Isolated from the lipoaspirate of healthy donors, ASCs were expanded in a basal growth medium before undergoing neural differentiation procedures. For this purpose, CM obtained from olfactory ensheathing cells and from Schwann cells were used. In some samples, 1 µM of melatonin was added. After 1 and 7 days of culture, cells were studied using immunocytochemistry and flow cytometry to evaluate neural marker expression (Nestin, MAP2, Synapsin I, GFAP) under different conditions. The results confirmed that a successful neural differentiation was achieved by glial CM, whereas the addition of melatonin alone did not induce appreciable changes. When melatonin was combined with CM, ASC neural differentiation was enhanced, as demonstrated by a further improvement of neuronal marker expression, whereas glial differentiation was attenuated. A dynamic modulation was also observed, testing the expression of melatonin receptors. In conclusion, our data suggest that melatonin’s neurogenic differentiation ability can be usefully exploited to obtain neuronal-like differentiated ASCs for potential therapeutic strategies.

## 1. Introduction

In the adult organism, stem cells are found in specialized areas known as niches [1,2]. By their self-renewal and differentiation ability, they ensure lifetime tissue turnover and healing from injury or disease. Initially identified in bone marrow, mesenchymal stem cells (MSCs) have since been found in virtually all adult tissues [3]. They are characterized by their positivity to typical MSC markers (CD44, CD73, CD90, and CD105) [4] and by their ability to differentiate into mesodermal cell lines such as chondrocytes, adipocytes, and osteoblasts. However, under suitable culture conditions, MSCs can also trans-differentiate into various cell lines, including nerve cells [5,6,7,8]. Among the many tissue sources, for instance bone marrow, umbilical cords, and dental pulp, adipose tissue emerges as a valuable source of MSCs. In fact, adipose-derived stem cells (ASCs) can be readily isolated in large amounts, can be easily expanded and stored, and show low immunogenicity [9,10]. For these reasons, ASCs have been extensively studied in the field of regenerative medicine to develop cell-based therapeutic approaches in cases of human pathologies to counteract tissue damage or degeneration, as occur for nervous tissue diseases.

Neural-like differentiation of ASCs can be achieved by various strategies, most involving adding to the culture medium a number of bioactive molecules such as retinoic acid, valproic acid, butylated hydroxyanisole, β-mercaptoethanol, forskolin, N2, and B27 [7,11,12]. In previous studies, we showed that ASCs can assume a neural phenotype, similar to neural progenitor cells (NPCs), when they are cultured in conditioned media (CM) from glial cells such as olfactory ensheathing cells (OECs) or Schwann cells (SCs) [13,14]. This approach avoids the use of potentially toxic chemical substances and creates, to some extent, a more physiological microenvironment. In this case, ASCs’ neural differentiation is likely promoted by a plethora of cytokines and growth factors normally released by these glial cells.

OECs derive from the olfactory bulb, have a permanent function of neural regeneration, and secrete various factors such as platelet-derived growth factor, brain-derived neurotrophic factor (BDNF), nerve growth factor (NGF), neurotrophin-4/5, and others [15,16,17,18,19]. When transplanted into the injured spinal cords of rats, OECs improved spinal cord function, probably due to a neuroprotective effect [20,21,22]. Schwann cells are present in the peripheral nervous system, where they surround axons with myelin during postnatal development [23,24,25]. They also produce neurotrophic factors, many of which are similar to those secreted by OECs [26]. Schwann cells are involved in the development and growth of the axons of embryonic dopaminergic neurons [27]; they promote survival and regeneration of retinal ganglion cells [28,29].

In physiological conditions, NPC differentiation is also dynamically controlled by hormones acting as signaling molecules [30,31,32]. In a previous study, we explored the effect of ghrelin, a gastrointestinal hormone with neuromodulator activity, on ASC neural differentiation, and we found it enhances this process [33].

The aim of the present work was to test whether ASC neural differentiation can be influenced by the hormone melatonin. Also called 5-methoxy-N-acetyltryptamine, melatonin is mainly secreted by the pineal gland. It is involved in the regulation of the circadian rhythm [34] and has been shown to have effects on the proliferation and differentiation of NPCs in various studies [35,36,37]. In vivo evidence indicates that melatonin may improve hippocampal neurogenesis and preserve memory function in mice [38]. It acts through melatonin receptors (MT) that are ubiquitously distributed. They are largely subdivided into MT Type 1 or 1A (MT1 or MT1A) and MT Type 2 or 1B (MT2 or MT1B). They are part of the G protein-coupled receptor family [39,40]. In 2004, Niles et al. [37] found MT receptors in NPCs, suggesting an involvement of this pleiotropic hormone in mammalian neurodevelopment. An improved endogenous neurogenesis and neural functions through MT2 receptors were also observed in an ischemic mouse model [41].

Herein, melatonin’s effects on ASC neural differentiation were tested in vitro using various strategies: by either adding the hormone to the basal culture medium or by testing possible synergistic effects in combination with CM from glial cells (OECs or Schwann cells). Using immunocytochemistry and flow cytometry, neural differentiation was assessed by monitoring the expression of typical markers such as Nestin, microtubule associate protein 2 (MAP2), Synapsin I, and the glial marker glial fibrillary acidic protein (GFAP) [13,14]. Immunostaining for melatonin receptors MT1 and MT2 was also investigated.

## 2. Results

### 2.1. ASC Viability

**Figure 1:**
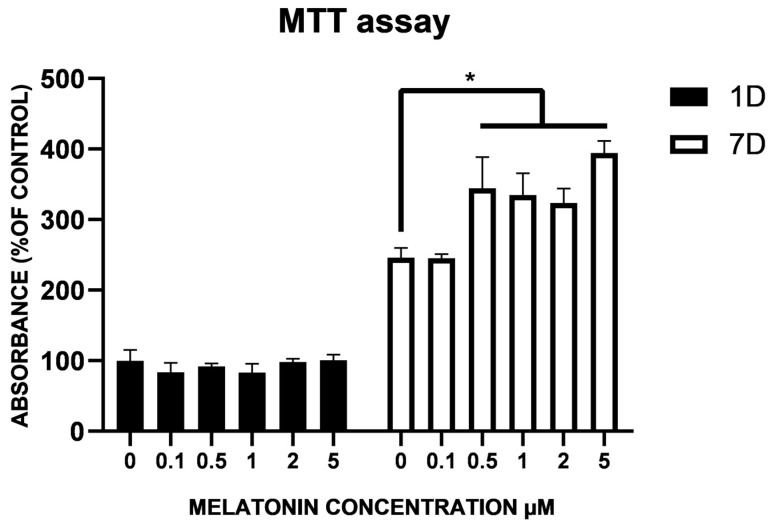
Effects of increasing concentrations of melatonin on ASC viability. MTT assay on day 1 (black bars) and 7 (white bars) of culture show that no toxic effect is exerted by melatonin at the concentrations tested (0.1 to 5 µM) as compared with controls (0 µM). An increased proliferation rate of adipose-derived mesenchymal stem cells (ASCs) can be observed on day 7 for melatonin concentrations of 0.5 µM and above. Absorbance values were determined at 570 nm. Values are expressed as mean ± SD of three independent experiments. All values are referred to control ASCs after 1 day of culture. * *p* < 0.05 vs ASCs at the corresponding day of culture.

We first evaluated the potential toxic effects of melatonin on cell viability by MTT (3-[4,5-dimethylthiazol-2-yl]-2,5 diphenyl tetrazolium bromide) assay. To this purpose, increasing concentrations of the hormone were tested on samples of ASC cultures. Cells were grown in the absence or in the presence of 0.1, 0.5, 1, 2 and 5 µM melatonin and tested after 1 and 7 days of culture (Figure 1). On day 1, ASCs exposed to the hormone did not show significant changes at any tested concentrations when compared with control ASCs, which were simply cultured in their basal growth medium. Similar results were also observed after 7 days, indicating that melatonin did not exert toxic effects. In particular, at concentrations of 0.5 µM and above, an increased proliferative effect was visible.

Melatonin 1 µM was chosen for the experiments because the data available in the literature show that this concentration enhances the differentiation capacity of NPCs [35,42,43] and MSCs [44]. On the other hand, concentrations of 5–100 µM melatonin have been reported to have antioxidant effects [45,46]. Therefore, higher concentrations were discarded to avoid misleading mixed effects.

In these experimental procedures, 1µM melatonin was added to ASCs, both in the basal growth medium and in cultures where the basal medium was replaced by OEC-CM or SC-CM. Consequently, six experimental groups were tested: (1) control ASCs in their basal medium (ASCs); (2) ASCs supplemented with melatonin (ASCs + M); (3) ASCs cultured in OEC-CM (ASCs/OEC-CM); (4) ASCs cultured in OEC-CM supplemented with melatonin (ASCs/OEC-CM + M); (5) ASCs cultured in SC-CM (ASC/SC-CM); and (6) ASCs cultured in SC-CM supplemented with melatonin (ASC/SC-CM + M).

### 2.2. Cell Morphological Features

**Figure 2:**
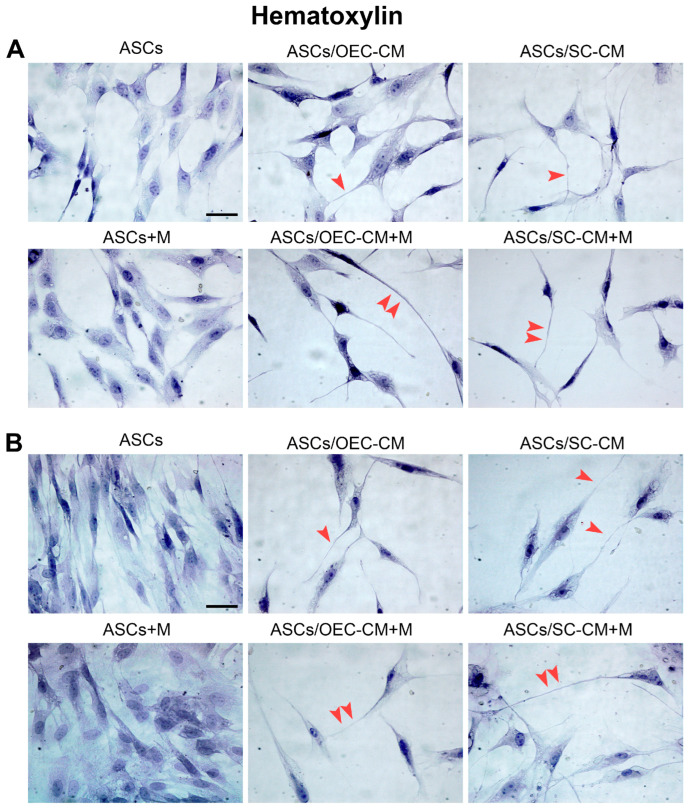
Hematoxylin staining of ASC cultures after 1 day (**A**) and 7 days (**B**) of growth in different conditions. Control ASCs and those grown in a conditioned medium from OECs and SCs are illustrated in the top row of each panel. In corresponding samples, 1 μM melatonin was added (bottom row of each panel). It can be stated that, regardless of the presence of melatonin, denser cell populations are present after 7 days of culture if they are kept in their basal medium, in which they show the typical fibroblast-like shape (first column). Less dense populations were observed in the presence of glial conditioned media (second and third column), especially on day 7. In these conditions, more elongated cytoplasmic prolongations can be observed (arrowheads), especially in the presence of melatonin (double arrowheads).

As previously reported [14], a typical fibroblast-like morphology was observed in control ASCs after 1 day of culture in the basal growth medium. The same shape was also detected on day 7, when a denser population was present (Figure 2). In these conditions, the addition of melatonin did not induce visible modifications. When cultured with OEC-CM or SC-CM, a lower proliferation rate was observed on both day 1 and 7, and cells exhibited a more complex morphology, featuring long cytoplasmic branches (arrows). This neural-like morphology was made more pronounced by the addition of melatonin (double arrows).

### 2.3. Expression of Neural Markers in ASCs

The expression of typical neural markers was evaluated by immunofluorescence and flow cytometry in ASCs undergoing different treatments, after 1 and 7 days of culture. In particular, Nestin, MAP2, Synapsin I, and the glial marker GFAP were investigated. Nestin is typical of neural stem/progenitor cells. MAP2 and Synapsin I are considered mature neuron markers. GFAP is typical of glial cells.

#### 2.3.1. Nestin Expression

**Figure 3:**
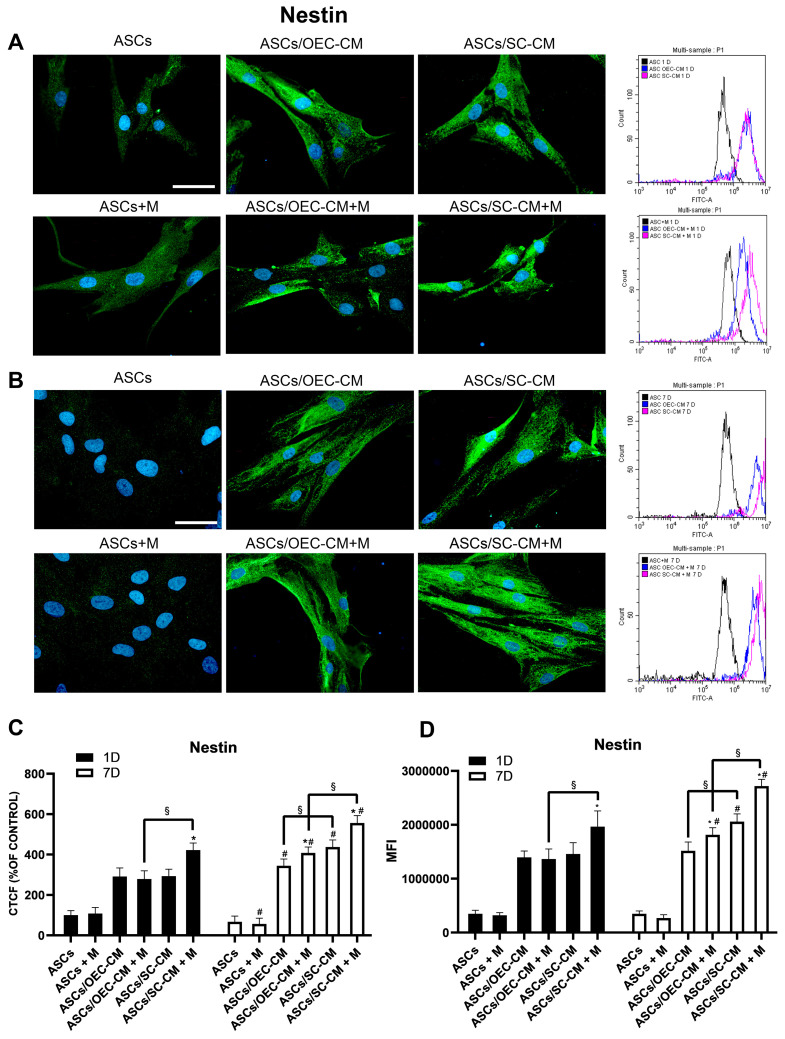
Nestin expression in ASC cultures. The different conditions were investigated by immunocytochemistry and flow cytometry on day 1 (**A**) and 7 (**B**) of culture. In each row, microphotographs are followed by flow cytometry histograms (fourth column) in the same conditions. Quantitative immunofluorescence data (ImageJ) and flow cytometry mean fluorescence intensity (MFI) are also reported in panels (**C**,**D**), respectively. A weak Nestin expression is detected when ASCs are grown in their basal medium, after both 1 and 7 days, regardless of the presence of melatonin (first column). A substantial increase is, however, observed when glial CM are used (second and third columns). Blue fluorescence counterstaining indicates cell nuclei. Scale bars: 50 µm. Quantitative data reported in the histograms (panels **C**,**D**) show that on day 1 the addition of melatonin is able to increase Nestin expression only in combination with SC-CM. On day 7, a more pronounced increase can be noticed for the same combination, although a modest increase is also present for the combination melatonin/OEC-CM. Each plot in panel (**C**) summarizes a total of 60 measurements. * *p* < 0.05 melatonin presence vs. correspondent melatonin absence; # *p* < 0.05 day 7 vs. day 1; § *p* < 0.05 OEC-CM vs. SC-CM with or without melatonin.

Immunocytochemical and flow cytometry data show that a weak Nestin expression was detected on day 1 and 7 in control ASCs and when melatonin was added (Figure 3). An increase in Nestin expression was observed following glial CM treatments, showing more marked increases on day 7 when using SC-CM. The presence of melatonin was able to increase Nestin expression on day 1 only when combined with SC-CM. An increased Nestin expression was noted in combination with both media on day 7, particularly with SC-CM. Overall, our results would indicate that melatonin improves neural ASC differentiation in combination with glial CM.

#### 2.3.2. MAP2 Expression

**Figure 4:**
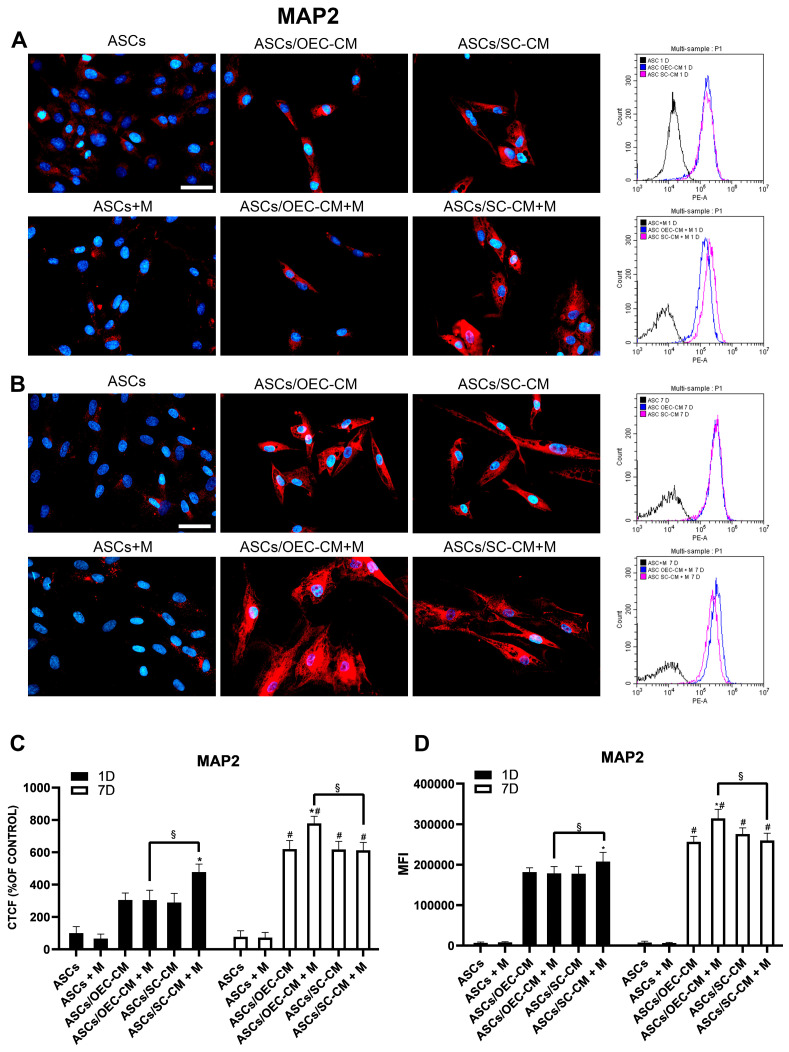
MAP2 expression in ASC cultures. The different conditions were investigated by immunocytochemistry and flow cytometry after 1 (**A**) and 7 (**B**) days of culture. In each row, microphotographs are followed by flow cytometry histograms (fourth column) in the same conditions. Quantitative immunofluorescence data (ImageJ) and flow cytometry mean fluorescence intensity (MFI) are also reported in panels (**C**,**D**), respectively. A virtually absent MAP2 expression is detected when ASCs are grown in their basal medium, after both 1 and 7 days, regardless of the presence of melatonin (first column). On day 1, a significant increase is, however, observed when glial CM are used, especially when melatonin was added to SC-CM. More pronounced increases were induced on day 7 by glial CM; in this case they were particularly evident for the combination melatonin/OEC-CM. Blue fluorescence counterstaining indicates cell nuclei. Scale bars: 50 µm. Quantitative data reported in the histograms (panels **C**,**D**) confirm that on day 1, the addition of melatonin is able to increase MAP2 expression only in combination with SC-CM; on day 7, a more pronounced increase can be noticed for the combination melatonin/OEC-CM. Each plot in panel (**C**) summarizes a total of 60 measurements. * *p* < 0.05 melatonin presence vs. corresponding melatonin absence; # *p* < 0.05 day 7 vs. day 1; § *p* < 0.05 OEC-CM vs. SC-CM with or without melatonin.

Immunocytochemical and flow cytometry data show that MAP2 expression was almost undetectable in control ASCs on both day 1 and 7, regardless of melatonin addition (Figure 4). Both CM treatments enhanced MAP2 expression, with the effect showing more strongly on day 7. The combination with melatonin produced varied effects. It improved SC-CM-induced increases on day 1 and improved OEC-CM-related increases on day 7.

Since MAP2 is involved in neuronal microtubule organization, its increase can explain the greater length of ASC cytoplasmic elongations observed when melatonin was combined with glial conditioned media in the cultures (double arrows in Figure 2).

#### 2.3.3. Synapsin I Expression

**Figure 5:**
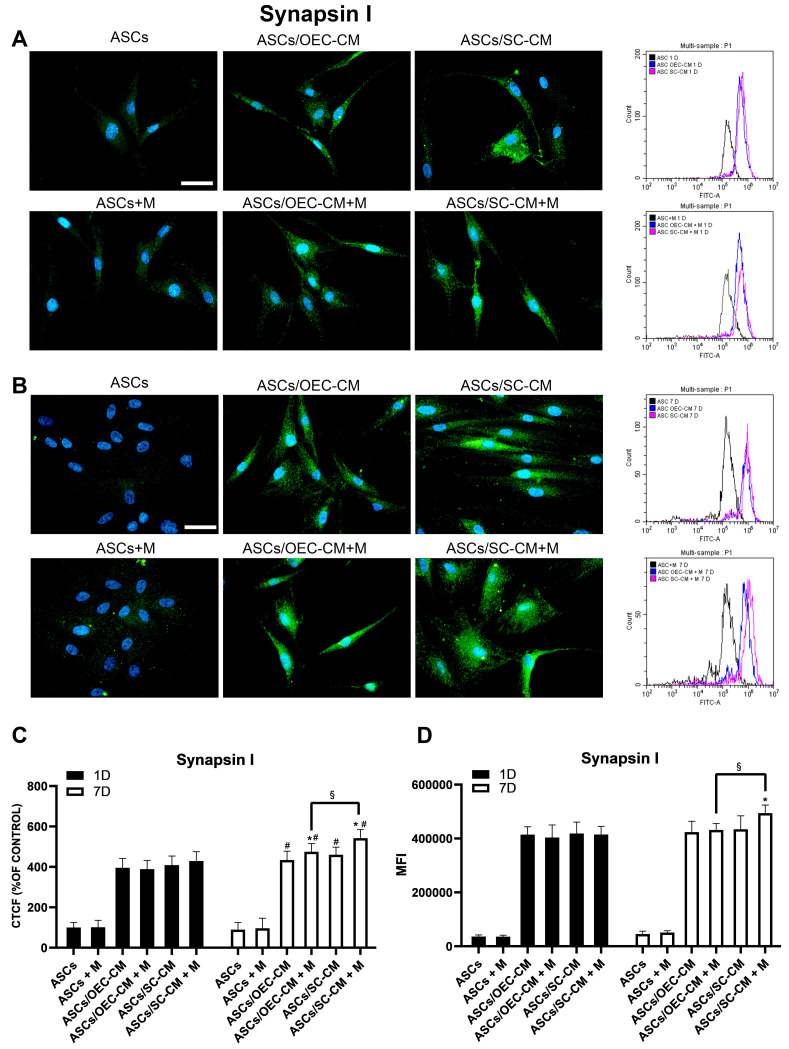
Synapsin I expression in ASC cultures. The different conditions were investigated by immunocytochemistry and flow cytometry after 1 (**A**) and 7 (**B**) days of culture. In each row, microphotographs are followed by flow cytometry histograms (fourth column) in the same conditions. Quantitative immunofluorescence data (ImageJ) and flow cytometry mean fluorescence intensity (MFI) are also reported in panels (**C**,**D**), respectively. A virtually absent Synapsin I expression is detected when ASCs are grown in their basal medium, on both day 1 and 7, regardless of the presence of melatonin (first column). On day 1, a significant increase is observed when glial CM are used (second and third column), with no evident effects when melatonin was also present. Comparable increases were induced on day 7 by CM treatments; a greater increase was obtained using the combination melatonin/SC-CM. Blue fluorescence counterstaining indicates cell nuclei. Scale bars: 50 µm. Quantitative data reported in the histograms (panels **C**,**D**) confirm Synapsin I increases following glial CM. A melatonin-induced increase on day 7 in combination with SC-CM is also confirmed. Each plot in panel (**C**) summarizes a total of 60 measurements. * *p* < 0.05 melatonin presence vs. corresponding melatonin absence; # *p* < 0.05 day 7 vs. day 1; § *p* < 0.05 OEC-CM vs. SC-CM with or without melatonin.

Immunocytochemical and flow cytometry data show that Synapsin I expression was almost undetectable in control ASCs on both day 1 and 7, regardless of melatonin addition (Figure 5). Also in this case, both CM treatments enhanced Synapsin I expression; the effect was detectable on day 1. Comparable increases were also present on day 7; at this time point, a further increase was produced by the addition of melatonin to SC-CM.

Since Synapsin I is a typical marker of the synaptic vesicles present in mature neurons, its improved expression is indicative of neuronal differentiation.

#### 2.3.4. GFAP Expression

**Figure 6:**
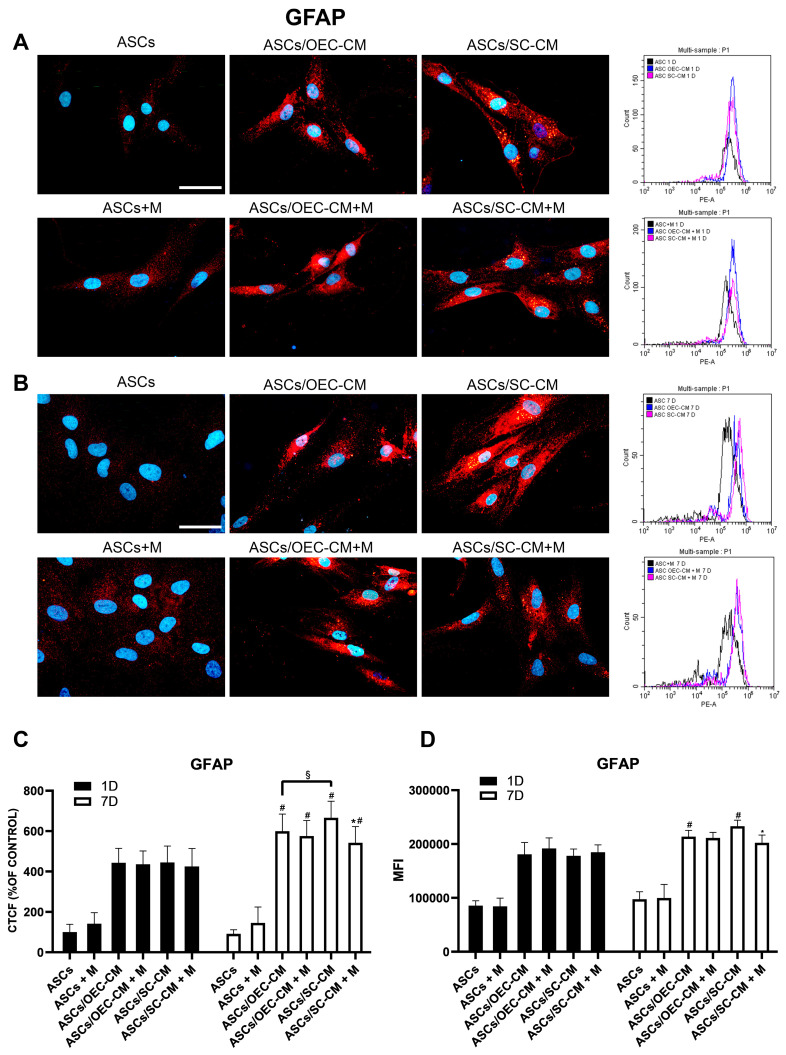
GFAP expression in ASC cultures. The different conditions were investigated by immunocytochemistry and flow cytometry after 1 (**A**) and 7 (**B**) days of culture. In each row, microphotographs are followed by flow cytometry histograms (fourth column) in the same conditions. Quantitative immunofluorescence data (ImageJ) and flow cytometry mean fluorescence intensity (MFI) are also reported in panels (**C**,**D**), respectively. A weak basal expression of GFAP is detectable when ASCs are grown in their basal medium, on both day 1 and 7, regardless of the presence of melatonin (first column). A significant increase is observed on both day 1 and 7 when glial CM were used (second and third column). The addition of melatonin to glial CM did not affect GFAP expression on day 1, whereas it attenuates CM-induced increases on day 7, especially in combination with SC-CM. Blue fluorescence counterstaining indicates cell nuclei. Scale bars: 50 µm. Quantitative data reported in the histograms (panels **C**,**D**) confirm the GFAP expression modifications above described. Each plot in panel (**C**) summarizes a total of 60 measurements. * *p* < 0.05 melatonin presence vs. corresponding melatonin absence; # *p* < 0.05 day 7 vs. day 1; § *p* < 0.05 OEC-CM vs. SC-CM with or without melatonin.

Immunocytochemical and flow cytometry data showed a low GFAP expression in control ASCs on both day 1 and 7, regardless of whether or not melatonin was added (Figure 6). Also in this case, both CM treatments strongly enhanced GFAP levels beginning on day 1. A further increase was observed on day 7, especially for the SC-CM treatment. However, the combination of glial CM with melatonin produced smaller increases, especially on day 7 when the hormone was added to SC-CM. This finding suggests that melatonin would negatively interfere with a glial ASC differentiation.

Overall, these results indicate that melatonin can accelerate the effects of SC-CM, being able to induce an early expression of some neuronal marker, and it also has positive effects on OEC-CM treatment, although at day 7 (a delayed effect). Moreover, melatonin attenuated the effects of CM on the expression of GFAP, suggesting a differentiation toward a neuronal rather than a glial lineage.

### 2.4. Expression of Melatonin Receptors in ASCs

Since melatonin interacts with its receptors in the nervous system, the expression of membrane MT1 and MT2 receptors was here investigated under different conditions.

#### 2.4.1. Melatonin Receptor MT1

**Figure 7:**
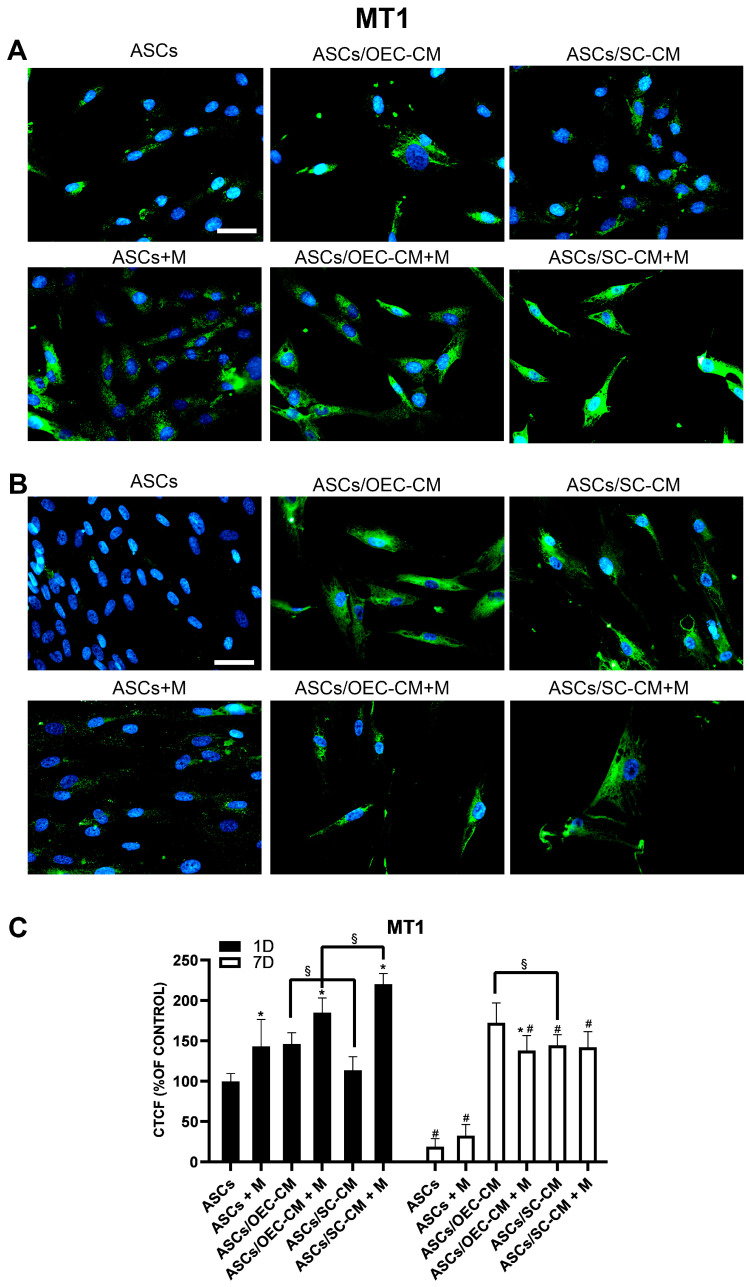
MT1 expression in ASC cultures. The different conditions were investigated by immunocytochemistry after 1 (**A**) and 7 (**B**) days of culture. Quantitative immunofluorescence data (ImageJ) are reported in panel (**C**). The basal MT1 expression detectable on day 1 in control ASCs almost disappears on day 7, showing at both time points an increase after melatonin addition. OEC-CM treatment also increased MT1 expression, especially on day 7; in combination with melatonin, a further increase was obtained on day 1, whereas lesser increases were observed on day 7. On the other hand, SC-CM treatment produced no significant MT1 expression modifications on day 1, whereas an increase was observed on day 7; in combination with melatonin, a robust increase in MT1 expression was visible on day 1, whereas no appreciable modification was detected on day 7. Blue fluorescence counterstaining indicates cell nuclei. Scale bars: 50 µm. Quantitative data reported in the histogram of panel (**C**) confirm MT1 expression modifications above described. Each plot in panel (**C**) summarizes a total of 60 measurements. * *p* < 0.05 melatonin presence vs. corresponding melatonin absence; # *p* < 0.05 day 7 vs. day 1; § *p* < 0.05 OEC-CM vs. SC-CM with or without melatonin.

Immunocytochemical data show that the basal ASC expression of the MT1 receptor on day 1 was slightly enhanced by the addition of melatonin (Figure 7). OEC-CM treatment produced a significant increase in MT1 expression, further improved in the presence of melatonin. While no effect could be observed for the treatment with SC-CM alone, the combination with melatonin strongly enhanced MT1 expression. On day 7, a dramatic reduction of MT1 expression was detected in control ASCs and in the presence of melatonin. At the same point in time, strong OEC-CM-induced increases were lessened by the addition of melatonin. On the other hand, substantial SC-CM-induced increases were not apparently modified in combination with the hormone.

It can be inferred that the MT1 receptor is downregulated over time in control ASCs, and that melatonin mitigates its expression when it is raised by CM treatment.

#### 2.4.2. Melatonin Receptor MT2

**Figure 8:**
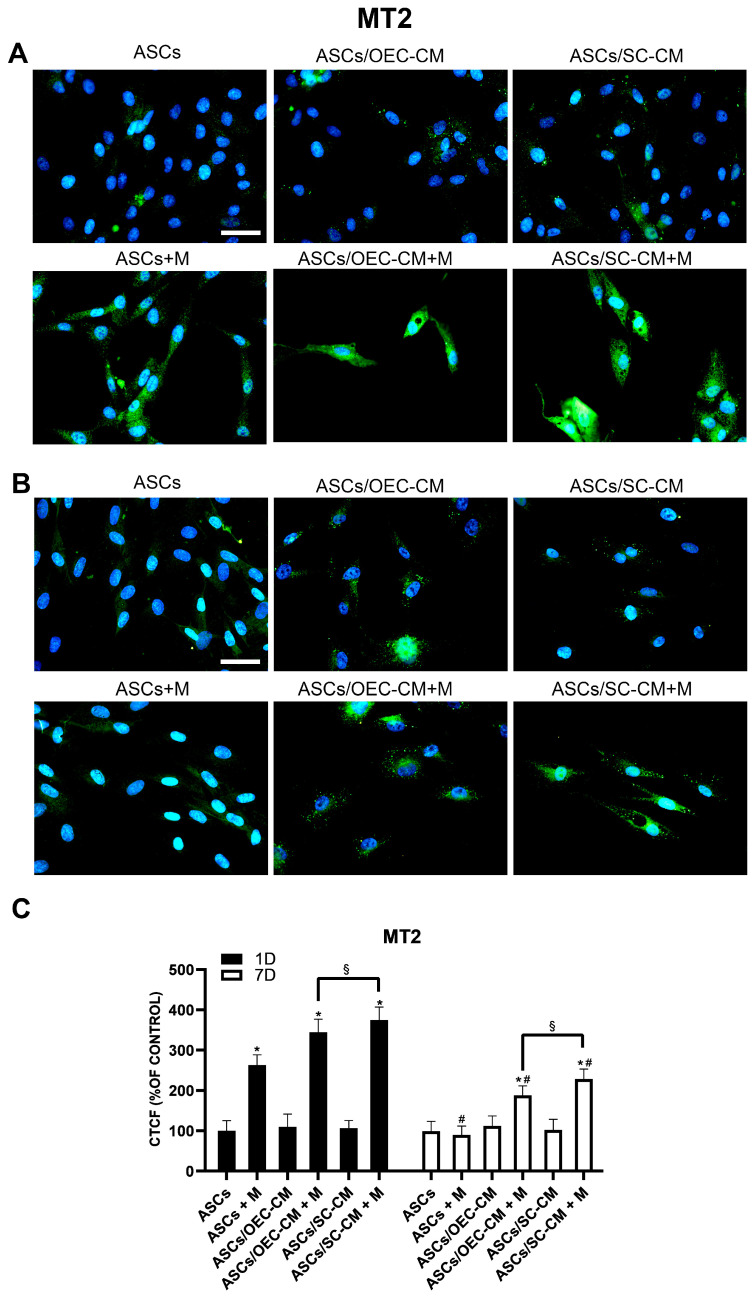
MT2 expression in ASC cultures. The different conditions were investigated by immunocytochemistry after 1 (**A**) and 7 (**B**) days of culture. Quantitative immunofluorescence data (ImageJ) are reported in panel (**C**). The basal MT2 expression detectable on day 1 when ASCs are grown in their basal medium is significantly increased when melatonin is present, whereas the same basal levels are unmodified on day 7, notwithstanding melatonin. At both detection times, no evident changes could be obtained following both CM treatments. In combination with melatonin, a significant enhancement of MT2 expression was observed, especially on day 1. Blue fluorescence counterstaining indicates cell nuclei. Scale bars: 50 µm. Quantitative data reported in the histogram of panel (**C**) confirm the MT2 expression modifications above described. Each plot in panel (**C**) summarizes a total of 60 measurements. * *p* < 0.05 melatonin presence vs. correspondent melatonin absence; # *p* < 0.05 day 7 vs. day 1; § *p* < 0.05 OEC-CM vs. SC-CM with or without melatonin.

Immunocytochemical data show that the basal ASC expression of the MT2 receptor on day 1 was significantly enhanced by the addition of melatonin (Figure 8). While neither CM treatment produced significant effects when applied alone, an improved MT2 expression was elicited by combining them with melatonin. On day 7, no evident differences in the basal levels of MT2 expression were detected in the control ASCs. In this case, no change was induced by the presence of melatonin. Moreover, melatonin addition increased MT2 expression levels, which were largely unmodified by CM treatment alone. It can be inferred that the MT2 receptor is upregulated soon after melatonin exposure, but the effects become less evident over time.

Overall, an increased expression of MT1 and MT2 receptors was obtained after melatonin treatment. The increased expression was particularly evident at early stages. CM treatment alone was able to increase MT1 expression with longer exposure, whereas no evident changes could be observed for MT2 expression. In combination with melatonin, greater increases were again observed at early observations, especially for SC-CM treatment. After a longer exposure time, no significant effects (MT1) or fewer increases (MT2) were observed.

## 3. Discussion

In the present work, the effects of melatonin on ASC neural differentiation were examined, as numerous studies in the literature have highlighted the hormone’s significant role in promoting neuronal differentiation. Specifically, melatonin has been shown to regulate various processes including the survival, proliferation, and neuronal differentiation of NPCs, as well as the survival and maturation of newly differentiated neurons [47].

Initial experiments had already showed that no significant effects can be produced by the mere addition of melatonin to the culture medium, probably because ASCs show a lower neural differentiative potential when compared to NPCs. However, it was also reported that more pronounced effects were observed in favorable microenvironments, where melatonin may act as an important regulator of precursor cells toward a neuronal commitment [48,49]. For this reason, we tested melatonin’s effects in combination with other neural differentiation strategies, such as glia-derived CM.

Our results confirmed that a neural ASC differentiation can be obtained by using glial conditioned media, as previously reported [13,14]. In fact, an overexpression of all tested markers was consistently observed. Moreover, it was confirmed that both neuronal (i.e., MAP2 and Synapsin I) and glial (i.e., GFAP) markers were overexpressed, suggesting that neural-like ASCs might still be in the early stages of differentiation. In fact, it has been reported that in NPCs, both neuronal and glial markers still coexist [50,51,52]. In agreement with this interpretation, Nestin, a typical NPC marker, was also overexpressed.

Nestin is an intermediate filament protein that plays a mandatory role for the early stages of NPC differentiation, either toward a neuronal or glial fate. As the differentiation progresses, a neuronal or glial fate would be overall favored, presumably on the basis of microenvironmental cues. Not only is it expressed in migrating and proliferating cells during embryogenesis, but it can also be found within regeneration areas in adult tissues [53]. Its increased expression may be indicative of an improved proliferative capacity. Furthermore, Nestin represents a characteristic marker for a multipotential differentiation [54]. However, its co-expression with lineage-specific markers is indicative of a given commitment before terminal differentiation, when Nestin expression is downregulated. Melatonin-induced overexpression of Nestin is suggestive of a role of the hormone in promoting neural differentiation. Therefore, our findings suggest that early Nestin expression may be associated with the initial steps of ASC neural differentiation. We found that an increase in Nestin was particularly evident when melatonin was combined with SC-CM.

The increased expression of MAP2 would be indicative of a melatonin-induced preferential neuronal differentiation. In fact, MAP2 contributes to microtubule stabilization, which is fundamental to regulate cell migration and division in neurons, to modify and maintain cytoplasmic elongations and cellular morphology, and to guide intracellular trafficking. It is expressed shortly after the switch from neuronal precursor to neuron. Predominantly located in the somato-dendritic compartment, it can also be detected in axons, where it enhances neurite growth [55]. High MAP2 levels might be responsible for the reduced proliferation rate as differentiation goes on [56,57]. MAP2 increases observed after melatonin-CM treatment may explain the more pronounced thin cytoplasmic prolongation observed in our ASC cultures, especially for the combination melatonin-OEC-CM. This is not surprising, since MAP2 is expressed in the olfactory system, where it is involved for the lifetime of the individual in the growth and differentiation of sensory neurons. Moreover, melatonin treatment was able to increase the percentage of MAP2-positive cells in cultures of NPCs [36].

The increased expression of Synapsin I, especially when melatonin was combined with SC-CM, also supports a preferential neuronal differentiation. In both the central and peripheral nervous systems, Synapsin I is expressed only in neurons, preferentially concentrated in presynaptic terminals, where it is associated with synaptic vesicles [58]. Although it is reported that Synapsin I synthesis mainly occurs when an innervating axon reaches its target neuron, it can also happen in the absence of cell-cell interaction, as a genetically programmed event to develop at the synaptic contact. Synapsin I would also be associated with elongation of cytoplasmic branches [59].

Interesting results were obtained testing GFAP expression modifications following melatonin/CM treatments. In agreement with our previous findings, it was confirmed that a substantial increase can be obtained with OEC-CM and, especially, with SC-CM, suggesting an evident trend toward a glial commitment. In fact, GFAP is a typical intermediate filament mainly expressed by astrocytes. However, although melatonin alone was not able to induce significant modifications, when added to glial CM, it generally attenuates CM-induced increases, particularly in combination with SC-CM. These effects are in counter-tendency with those obtained from neuronal markers, leading us to hypothesize once again that melatonin sustains a neuronal commitment. Indeed, numerous data existing in the literature support an involvement of melatonin toward a neuronal differentiation. In neural stem cell lines, a melatonin-related increase in neuronal marker expression (Nestin and Tubulin β3) was observed, along with a decrease in GFAP positive cells and mRNA levels for this protein [36]. In amniotic fluid MSC cultures, a neurogenic medium supplemented with melatonin was able to improve typical neuronal markers, such as Tubulin β3, MAP2 and tyrosine hydroxylase, and to decrease GFAP levels [44].

Immunostaining for the melatonin receptors MT1 and MT2 was carried out, since these membrane receptors seem involved in NPC neurodifferentiation [60]. In fact, although expressed in several adult tissues, they are diffusely expressed in the central nervous system [36]. MT1 is found particularly in the suprachiasmatic nucleus, paraventricular nucleus, and area postrema. MT2 is more expressed in the hippocampus, substantia nigra, and ventral tegmental area than in the suprachiasmatic nucleus. In our results, MT1 and MT2 levels were differentially modulated in the different culture conditions. Their early expression, revealed on day 1, seemed to be improved by the addition of melatonin in all conditions, although a decrease was observed over time, even in the presence of the hormone. MT1 levels also increased following CM treatments. In fact, OEC-CM alone was able to improve MT1 expression beginning on day 1 and SC-CM on day 7. Since Schwann cells express MT receptors [61], it can be hypothesized that mRNA encoding for MT1 is present in their CM and it might be internalized, translated, and exposed at the membrane level. A similar effect might explain the OEC-CM-induced effects. In fact, the presence of mRNAs encoding for MT receptors were previously reported in the olfactory bulb [62]. However, other molecules not yet identified in glial CM could play a role in MT expression.

Different effects evoked by OEC-CM and SC-CM can be explained by a difference in the various cytokine/neurotrophic factors secreted. Recent studies suggest that, although both OECs and Schwann cells produce NGF and BDNF, glial cell line-derived neurotrophic factor (GDNF) is released by OECs, whereas ciliary neurotrophic factor and basic fibroblast growth factor are secreted by Schwann cells [63]. This might account not only for the different effects observed in this work, but also in previous studies [13,14,64]. In particular, SC-CM would induce a preferential glial commitment, as indicated by the increased GFAP expression, whereas OEC-CM would rather favor a neuronal differentiation, as indicated by increased levels of typical neuronal markers. In this respect, it should be noted that melatonin-induced modifications reinforce CM-induced neuronal marker expression, attenuating increased GFAP levels. This may be due to an increased expression of BDNF and GDNF, as occurs for NPCs [36,44].

Melatonin-induced neurogenic effects in NPCs and MSCs can be mediated by MT1 and MT2 by activating phosphoinositide 3-kinase (PI3K) and protein kinase B (Akt) signaling pathway. However, other mechanisms may also be considered, such as a receptor-independent increase in BDNF, which can act through the activation of the tropomycin-receptor kinase B (TrkB) signaling pathway [47,65]. In addition, epigenetic mechanisms, with or without MT receptor involvement, might also be responsible. In fact, melatonin-induced histone acetylation not only promotes neuronal differentiation, but also blocks a glial commitment [36]. Finally, emerging hypotheses underline a potential role of miRNA in controlling a stem cell’s fate, and it can be involved in the synergic effects of melatonin. Recent studies report a melatonin-related modulation of miRNA expression, which are involved in neurogenesis (let-7a and miR-124) [36].

Overall, our results indicate that melatonin can represent a valuable tool to differentiate ASCs into neuronal cells if used in combination with other strategies. This is supported by studies in which melatonin pre-treated ASCs decreased amyloid formation and improved learning, memory, and cognitive functions in a rat model of Alzheimer’s disease [66]. However, melatonin pre-treated ASCs were implanted in a rat model of spinal cord injury and tested for their neuronal differentiation, but no differences were found between native and pre-treated ASCs [67]. This is in agreement with the present results, showing that melatonin alone has no evident effects on ASC neural differentiation. Therefore, it can be assumed that a neuronal ASC pre-differentiation would be more efficacious in cell-based therapies if melatonin is combined with other neuroinductive strategies. Further studies would be useful to better clarify the molecular mechanisms involved in melatonin-induced neurogenic effects. This is of particular interest because ASCs are easily harvested compared to NPCs, making them excellent candidates for in situ transplantations. Moreover, due to their low immunogenicity, they can also be used for allogenic applications.

## 4. Materials and Methods

### 4.1. ASC Cultures

ASCs were obtained from adipose tissue harvested through abdominal liposuction procedures at the Cannizzaro Hospital (Catania, Italy), following a protocol approved by the local ethics committee (Comitato etico Catania1; Authorization n. 398/202l/EMPO), in accordance with the Declaration of Helsinki, and after the donors signed informed consent to use the lipoaspirate for experimental procedures. The donors were three healthy females (32–38 years old), non-smokers and occasionally taking non-steroidal anti-inflammatory drugs. The lipoaspirate (50–100 mL) was washed with sterile phosphate-buffered saline (PBS; Invitrogen) and then incubated at 37 °C with 0.075% of type I collagenase (Invitrogen) dissolved in serum-free low glucose Dulbecco’s modified Eagle’s medium (DMEM). After 3 h, collagenase was inactivated by adding an equal volume of DMEM and 10% fetal bovine serum (FBS, Sigma-Aldrich). Following various washing and centrifugation cycles (1200 rpm for 10 min) to remove debris, pellets were resuspended in PBS, and the cells were filtered through a 100-μm nylon cell strainer (Falcon BD Biosciences, Milan, Italy). Finally, they were seeded onto 75 cm^2^ flasks (Falcon BD Biosciences) with complete DMEM containing 10% FBS, 1% penicillin/streptomycin, 1% MSC growth supplement (MSCGS; ScienCell Research Laboratories, Milan, Italy) and incubated at 37 °C with 5% CO_2_. At confluence (about 80% of total flask surface), all cultures were trypsinized and cells were expanded for 3 passages before the subsequent procedures.

At first, some ASC samples were used to verify their MSC nature as previously reported [36]. In fact, they were immunopositive for CD44, CD73, CD90, and CD105 (commonly recognized as MSC markers) and immunonegative for typical hematopoietic stem cell markers (CD14, CD34, and CD45). Then, their ability to differentiate into cells of the mesodermal lineage (adipocytes, chondrocytes, and osteocytes) was ascertained.

To test melatonin toxicity, some ASC samples were used for the viability assay in basal conditions and after hormone addition. Some other samples underwent neural differentiation by using glial conditioned media, with or without melatonin addition.

### 4.2. ASC Viability by MTT Assay

ASC viability in the various conditions was evaluated by the MTT assay. Some control ASCs were compared with other samples in which melatonin was added at different concentrations (0.1 µM, 0.5 µM, 1 µM, 2 µM, 5 µM). Detection was carried out after 1 and 7 days to evaluate possible cytotoxic effects.

Briefly, about 8000 cells/well were seeded in 96-well microplates and incubated at 37 °C in a humidified atmosphere containing 5% CO_2_. At each detection point, 20 μL of 0.5% MTT in PBS was added and, after a further 3 h of incubation, the supernatant was removed and replaced with 100 μL of DMSO. The optical density of each sample was measured using a Multiskan SkyHigh Microplate spectrophotometer (Thermo Scientific) at λ = 570 nm. For each of the three cell lines, each sample was tested in quintuplicate. The ASC viability was calculated as: (optical density sample/average optical density control) × 100.

### 4.3. Glial Conditioned Media Preparation

As previously described [8,68], glial cells were isolated from 2-day old mouse pups according to the Italian Guidelines for Animal Care (D. Lgs 26/2014) and the European Communities Council Directives (2010/63/EU). Experimental procedures were approved by the ethics committee of the University of Catania (Organismo Preposto al Benessere Animale, OPBA; authorization n. 174/2017-PR). Animal suffering was minimized, as well as the number of animals used.

#### 4.3.1. Preparation of OEC-CM

OECs were isolated from mouse olfactory bulbs, which were dissected at +4 °C in Leibowitz L-15 cold medium (Sigma-Aldrich, Milan, Italy). They were then digested in Minimum Essential Medium-Hepes (MEM-H, Sigma-Aldrich), with the addition of 2.5% trypsin (Sigma-Aldrich) and 0.1% collagenase (Invitrogen, Milan, Italy). To stop enzymatic activity, DMEM supplemented with 10% FBS was added. OECs obtained after centrifugation were resuspended in DMEM and seeded in flasks. After initial plating, the antimitotic agent cytosine arabinoside (10^−5^ M) was added for 24 h in order to reduce the number of dividing fibroblasts. Subsequently, cells were incubated at 37 °C with DMEM supplemented with 1% penicillin/streptomycin and their OEC nature was identified by immunocytochemistry using S-100 as a marker. OECs were expanded in further passages and their CM was collected 24–48 h from confluence, filtered to remove debris, aliquoted and stored at −20 °C until further use.

#### 4.3.2. Preparation of SC-CM

To isolate Schwann cells, mouse sciatic nerves were removed and kept in DMEM supplemented with 1% penicillin/streptomycin. They were then dissected in trunks, chopped into 1 mm segments, and digested with 0.1% collagenase and 2.5% trypsin in DMEM. The digested tissue was then filtered through a 150 µm nylon mesh and, after centrifugation, the Schwann cells were resuspended in fresh growth medium (DMEM/FBS). After the initial plating, the antimitotic agent cytosine arabinoside (10^−5^ M) was added for 24 h in order to reduce the number of dividing fibroblasts. Subsequently, cells were incubated at 37 °C with DMEM supplemented with 1% penicillin/streptomycin and their nature was identified by immunocytochemistry using S-100 as a marker. Schwann cells were expanded in further passages and their CM was collected 24–48 h from confluence, filtered to remove debris, aliquoted and stored at −20 °C until further use.

### 4.4. ASC Neural-like Differentiation

In the present work, the influence of 1 µM melatonin, alone or in association with OEC-CM or SC-CM, was tested on the neural differentiation of ASCs.

A total of six groups of ASC cultures were prepared: (1) control ASCs in their basal medium (ASCs); (2) ASCs supplemented with melatonin (ASCs + M); (3) ASCs cultured in OEC-CM (ASCs/OEC-CM); (4) ASCs cultured in OEC-CM supplemented with melatonin (ASCs/OEC-CM + M); (5) ASCs cultured in SC-CM (ASC/SC-CM); and (6) ASC cultured in SC-CM supplemented with melatonin (ASC/SC-CM + Mel). Each sample was tested on days 1 and 7 of each treatment.

One sample of each group was stained with hematoxylin to inspect cell density and cytomorphological changes. The other samples underwent immunofluorescence and flow cytometry to evaluate specific neural marker expression (Nestin, MAP2, Synapsin I, GFAP). Some samples of each group were also investigated using immunostaining to evaluate melatonin receptor (MT1, MT2) expression.

#### 4.4.1. Immunofluorescence

Immunocytochemical staining was carried out following a protocol previously described [8]. Cells were fixed with 4% paraformaldehyde, washed with PBS and incubated for 30 min in PBS containing 5% normal goat serum (Sigma-Aldrich, Burlington, MA, USA) for blocking non-specific sites, and 0.1% Triton (Sigma-Aldrich) for permeabilization. They were then exposed overnight at 4 °C to primary antibodies: rabbit anti-Nestin (1:100; Abcam, Cambridge, UK, NBP1-02419), mouse anti-MAP2 (1:100; BioLegend, San Diego, CA, USA, 801810), rabbit anti-Synapsin I (1:100; Abcam, Ab64581), mouse anti-GFAP (1:100; BD, 610566), rabbit anti-MT1 (1:100; Cusabio, Houston, TX, USA, CSB-PA780156), and rabbit anti-MT2 (1:100; Novus, Chesterfield, MI, USA, NLS932SS). The next day, cells were washed with PBS and incubated in the dark for 1 h at room temperature with secondary antibodies conjugated to different fluorochromes: FITC-conjugated goat anti-rabbit (1:500; Abcam, ab96899) or Cy3-conjugated goat anti-mouse (1:500; Abcam, ab96880). Finally, cell nuclei were counterstained for 10 min with DAPI.

Immunofluorescence was detected using a Leica DMRB Fluorescence Microscope and digital images were acquired by a computer-assisted digital camera (Leica, Deer Park, IL, USA, DFC 320). Immunofluorescence densitometric analyses were performed using the FIJI-ImageJ measurement tool (National Institutes of Health, Bethesda, MD, USA).

For each time point, six digital photomicrographs were randomly selected from each sample of each experiment (cell line). Since three cell lines were tested, 18 microphotographs were used for each group. Four cells were measured on average from each microphotograph until a total of 60 measurements (20 from each cell line) were reached for each computation. Values were derived from the average grayscale intensity. The integrated density, the cell area, and the mean fluorescence of the selected cells were estimated. The same procedure was applied to three different background areas around the selected cell. Finally, the corrected total cell fluorescence (CTCF) was calculated using the following equation: CTCF = integrated density—(cell area × background mean fluorescence). The CTCF (% of control) was calculated as: (CTCF sample/average CTCF control) × 100.

#### 4.4.2. Flow Cytometry

For flow cytometry analysis, samples of each group were trypsinized, fixed with 1% paraformaldehyde for 20 min at 4 °C, and permeabilized with 1% Triton for 5 min at 4 °C. After blocking non-specific sites with 1% BSA for 30 min, cells were incubated for 1 h at room temperature with primary antibodies: rabbit anti-Nestin (1:200; Abcam, NBP1-02419), mouse anti-MAP2 (1:200; BioLegend, 801810), rabbit anti-Synapsin I (1:200; Abcam, Ab64581), and mouse anti-GFAP (1:200; BD, 610566). Finally, cells were incubated for 1 h at room temperature in the dark with FITC-conjugated goat anti-rabbit (1:500; Abcam, ab96899) and PE-conjugated goat anti-mouse (1:500, Santa Cruz, Dallas, TX, USA, sc-3764). Quantitative data were collected using a DxFLEX flow cytometer (Beckman Coulter Life Sciences; Brea, CA, USA). 10,000 events were detected for each sample. Mean Fluorescence Intensity (MFI) values were calculated and recorded automatically. Data were processed using the software CytExpert and subsequently graphed.

## Data Availability

Data are contained within the article.

## Abbreviations

ASCs: Adipose-derived mesenchymal stem cells; CM: Conditioned medium; CTCF: Corrected total cell fluorescence; GFAP: Glial fibrillary acidic protein; M: Melatonin; MAP2: Microtubule-associated protein 2; MT1: Melatonin receptor 1; MT2: Melatonin receptor 2; OEC-CM: Olfactory ensheathing cell-conditioned medium; SC-CM: Schwann cell-conditioned medium.
